# High-Speed-Ventral-Plane Videography Identifies Specific Gait Pattern Changes in Cuprizone-Induced Demyelination in Mice

**DOI:** 10.3390/cells14130969

**Published:** 2025-06-24

**Authors:** Paula Giesler, Markus Kipp, Alexander Hawlitschka

**Affiliations:** Institute of Anatomy, Rostock University Medical Center, Gertrudenstraße 9, 18057 Rostock, Germany; paula.giesler@uni-rostock.de

**Keywords:** microglia, gait, multiple sclerosis, cuprizone, motor cortex, corpus callosum, caudate putamen complex

## Abstract

Gait disturbances are among the most prominent motor symptoms in multiple sclerosis (MS), yet their functional characterization in preclinical models remains limited. In this study, we used high-speed ventral plane videography (DigiGait™) to analyze locomotor behavior during 5 weeks of cuprizone-induced demyelination in 10 male C57BL/6 mice. Gait analysis revealed significant alterations in stride time (left front paw from 0.303 ± 0.01 s to 0.257 ± 0.007 s; *p* = 0.003), paw angle (right fore paw from −13.78 ± 0.928° to 5.456 ± 2.146°; *p* = 0.003), and midline distance (right hind paw from 1.889 ± 0.099 cm to 1.216 ± 0.096 cm; *p* = 0.013), particularly in the hind limbs. These behavioral impairments correlated with histopathological findings of reduced myelination and elevated microglial activation in motor-relevant brain regions, including the corpus callosum, caudate-putamen, and motor cortex. Notably, specific gait parameters showed strong correlations with the degree of demyelination, supporting their relevance as functional biomarkers. Our data demonstrate that high-resolution gait analysis provides a sensitive, non-invasive tool to monitor functional deficits in demyelinating models and may aid in evaluating therapeutic efficacy in future studies.

## 1. Introduction

Multiple sclerosis (MS) is a chronic neurological disease of the central nervous system (CNS) caused by inflammation that leads to myelin and neuronal damage. Although MS is widely regarded as an autoimmune disorder [1,2,3], its exact pathogenesis remains a subject of intense debate. Several factors have been implicated in disease onset and progression, including insufficient exposure to ultraviolet light, vitamin D deficiency, viral infections, obesity, and genetic susceptibility [2,4,5]. Depending on the neuroanatomical location of the lesion(s), various behavioral domains can be affected in MS patients including sensory loss, muscle weakness, spasticity, vision impairment, or ataxia. Beyond this, fatigue is frequently reported by the patients and is often the most disabling symptom [2,6,7,8].

The Expanded Disability Status Scale (EDSS) is the most widely used tool to assess disability in MS patients. It ranges from 0 to 10 in 0.5-point increments, with higher scores indicating greater disability. The score is derived from a neurologist’s clinical examination and reflects functional impairments across eight neuroanatomical systems, with particular emphasis on motor function. EDSS scores from 1.0 to 4.5 denote individuals who can walk unaided [9]. Additional measures of motor disability in MS include the Timed 25-Foot Walk, which evaluates ambulatory function [10], and the 9-Hole Peg Test, which assesses upper-limb coordination and manual dexterity [11].

Gait disturbances are among the most frequent motor deficits in MS. Up to 85% of patients report gait impairment during the disease course, and 50–80% exhibit objectively measurable deficits in balance and locomotion. Gait abnormalities often arise early and progress with disease duration [12,13,14]; approximately 50% of patients require walking aids within 15 years of diagnosis, and around 10% become wheelchair-dependent [13,15,16,17]. From a mechanistic point of view, contributors to gait dysfunction in MS can include spasticity, muscle weakness, and impaired coordination. Spasticity, present in the majority of patients to some degree, leads to increased muscle tone and stiffness, eventually resulting in a spastic gait characterized by reduced knee flexion and leg scissoring [18]. Muscle weakness, particularly in the lower limbs, might contribute to foot drop and reduced propulsion, eventually manifesting as slower walking speed and shorter step length. Beyond this, cerebellar dysfunction might induce ataxia and postural instability, eventually leading to a broad-based, unsteady gait [19]. Notably, up to 80% of MS patients develop some degree of ataxia [20,21]. Of note, MS-associated motor impairments eventually result in patients being unable to walk unaided and, thus, being dependent on a wheelchair [22,23,24].

To develop novel therapeutic strategies for MS, various animal models are employed, broadly categorized into autoimmune and non-autoimmune types. The most widely used autoimmune model is experimental autoimmune (EAE). In EAE, rodents, usually mice, are immunized with an antigen of a myelin-related protein present in the CNS, like recombinant myelin oligodendrocyte glycoprotein (MOG), MOG peptides, proteolipid protein (PLP) peptides, or myelin basic protein—typically in complete Freund’s adjuvant—triggering antigen uptake by professional antigen-presenting cells (i.e., Langerhans dendritic cells of the skin and perivascular macrophages) and subsequent activation of encephalitogenic Th1 and Th17 cells in lymphoid tissues. This immune cascade induces inflammation, predominantly in the spinal cord and cerebellum [25]. Another frequently applied model is the cuprizone model, which leads to metabolic oligodendrocyte degeneration and, in consequence, demyelination [26,27,28]. In this model, demyelination mainly occurs among other regions in the forebrain, the caudo-putamen and around the cerebellar nuclei [29,30,31], whereas the spinal cord is not affected [32].

Different platforms are available to study motor-behavior in small rodents, including the rotarod apparatus and the DigiGait™ (Mouse Specifics Inc., Framingham, MA, USA) platform. The rotarod apparatus consists of an accelerating rotating rod on which mice are placed to assess motor coordination and balance. Positioned at a height that discourages voluntary descent, the rod gradually increases in speed, and the primary outcome is the latency to fall, automatically, usually recorded by an integrated timer. In contrast, the DigiGait™ platform employs a transparent treadmill and a high-speed ventral video capture system to record paw placement and movement as animals walk at controlled speeds. The system analyzes a range of gait parameters, including stride length, stance and swing durations, paw area, and interlimb coordination, which are derived through automated digital footprint analyses by a software, allowing for objective and reproducible quantification of gait abnormalities. In recent studies, we demonstrated, using DigiGait™ high speed ventral plane videography during the early EAE development, that distinct gait abnormalities occur which are not evident when conventional behavioral analyses are performed [33]. Beyond this, we were able to show that in the cuprizone model, despite complete remyelination, motor-behavior is impaired [34].

In this study we asked whether in the cuprizone model acute demyelination is paralleled by gait abnormalities. To this end, acute demyelination was induced by a 5-week cuprizone intoxication, and gait patterns were analyzed before and during the intoxication period via DigiGait™ high speed ventral plane videography. In parallel, the extent of demyelination and glial activation was evaluated by immunohistochemistry to explore potential correlations between myelin damage and gait alterations.

## 2. Materials and Methods

### 2.1. Animals

Seven-week-old male C57BL/6 mice (*n* = 10) were obtained from Janvier Labs, Le Genest-Saint-Isle, France. Upon delivery, the mice were randomly assigned to cages by a blinded technical assistant. All mice were subjected to cuprizone intoxication and complex gait analyses (see below). A maximum of five animals were housed per individual cage (814 cm^2^ area). The animals were provided with ad libitum access to food and water and were kept in a temperature-controlled room (22 ± 2 °C) with a 12 h light/dark cycle. As stated, the experimental group initially included ten mice, but one was excluded from further analyses due to its refusal to walk on the treadmill.

Then, 35 days after the start of continuous cuprizone intoxication, the mice were euthanized, and their brain pathology was examined through histological analyses. To evaluate the extent of histological changes induced by the cuprizone intoxication and to compare brain and animal weights at the end of the study, an untreated control group of four mice was included for comparison. All experiments were formally approved by the State Animal Research Committee of Mecklenburg-Western Pomerania (LALLF M-V 7221.3-1-001-19).

### 2.2. Experimental Design

**Figure 1:**
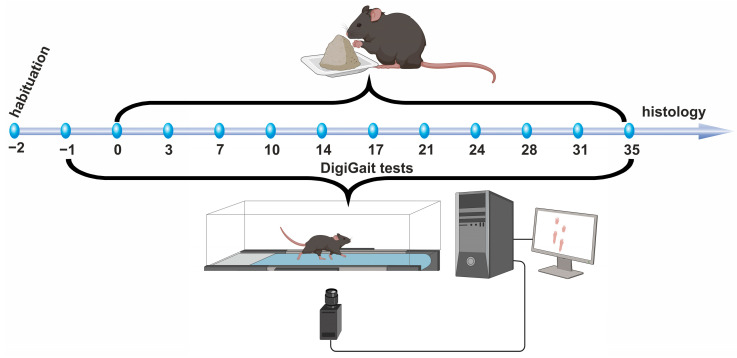
On day −2, mice were introduced to the DigiGait apparatus for habituation. The first gait measurement was conducted on day −1, prior to the initiation of cuprizone intoxication on day 0. A second gait measurement was performed on day 3, followed by biweekly testing over the subsequent five weeks. At the end of the five-week intoxication period, the mice were perfused, and histological analyses of their brains were performed.

Mice were introduced to the test apparatus for habituation one day before initiation of gait measurements (designated as day −2). To establish baseline values for gait parameters, the first gait analysis of the experimental mice was conducted on the day prior to initiation of the cuprizone intoxication (designated as day −1). Starting on day 0, the standard mouse diet was supplemented with 0.25% cuprizone (bis(cyclohexanone) oxaldihydrazone; Sigma-Aldrich, St. Louis, MO, USA) and provided in two separate Petri dishes per cage. Cuprizone intoxication was carried out following a standard protocol as previously described by our group [27,35,36]. Gait analyses were performed twice weekly. After five weeks, the mice were anesthetized via intraperitoneal injection of ketamine (100 mg/kg) and xylazine (10 mg/kg) and subsequently transcardially perfused. The brains were harvested, embedded in paraffin, and subjected to immunohistochemical analyses for PLP and anti-ionized calcium-binding adapter molecule 1 (IBA1) expression to label myelination and microglia densities, respectively. All experiments were conducted at the Institute of Anatomy, Rostock University Medical Centre. Mice were habituated on 26 February 2022. DigiGait testing was performed from 27 February to 4 April 2022. On 5 April 2022, the mice were perfused, followed by histological brain processing and densitometric analysis in the subsequent weeks. The experimental design is summarized in Figure 1.

### 2.3. High Speed Ventral Plane Videography and Evaluation

Gait parameters were assessed using the DigiGait™ imaging system along with the DigiGait™ 15.0 analysis software (Mouse Specifics, Inc.; Quincy, MA, MA) as previously published by our group [33,34]. The DigiGait™ apparatus consists of a clear plastic treadmill with a high-speed under-mounted digital camera (frames/s, Basler Technologies Inc., Ahrensburg, Germany) used for imaging paw prints during running. Animals were habituated to the machine 1 day prior to testing (compare Figure 1). The minimal duration of each video sequence required for subsequent footprint analyses was 5 s, which has been shown to be sufficient for reliable gait analyses in mice [37]. The velocity of the treadmill belt was 15 cm/s. Images were collected at a rate of 181 frames/s and stored as audio–video interleaved (AVI) files for later blinded analyses. To improve the contrast for automated footprint analysis, the tails of the mice were colored with black dye. The treadmill belt was cleaned with 70% (*v*/*v*) ethanol between each animal testing. The image analysis software digitally encoded the individual paw area and position relative to the tread-belt. Each paw of the animal was treated as a unique signature so that later analyses of foot movements could be performed on separate limbs. The DigiGait™ analysis software subsequently computed a variety of gait parameters for the fore limbs and for the hind limbs of each animal [33,34,37].

The DigiGait™ apparatus and accompanying software determined 39 gait parameters for the fore paws and 43 gait parameters for the hind paws. In this study, we presented the developments of stride time, stance phase, brake phase, propel phase, midline distance, swing time, and paw angle for both fore and hind paws. Furthermore, the development of the overlap distance was presented in this study; due to the nature of this parameter, it could only be determined for the left and right hind paws. The developments of the stance width and the sum PawAngle between the fore paws and between the hind paws were also presented in this study.

The characteristics of the gait parameters presented are briefly described below.

- Stride time

Stride time refers to the time it takes for a paw to complete a full gait cycle, from the initial contact of a paw to the next initial contact of the same paw. It is an important temporal parameter for evaluating gait patterns and assessing locomotor function.

- Stance phase

The stance phase refers to the portion of the locomotion cycle during which the paw is in contact with the ground, supporting the mouse’s body weight. It begins when the paw touches the ground (paw strike) and ends when the paw lifts off (paw-off).

- Brake phase

The brake phase refers to the portion of the stance phase where the paw applies decelerating forces against the ground, typically to reduce forward momentum or control movement. It occurs immediately after paw contact and precedes the subsequent propulsion phase.

- Propel Phase

The propel phase is the portion of the stance phase where the paw generates accelerating forces against the ground, pushing the body forward. It follows the brake phase and ends with the paw’s lift-off.

- Midline Distance

The midline distance refers to the distance between the center of each of the paws at maximum ground contact during a stride and the median horizontal plane of the animal’s body.

- Overlap Distance

Overlap distance refers to the horizontal distance between the placement of the fore paw and the hind paw on the same side during a step cycle.

- Stance width

Stance width refers to the vertical distance between the paws of the same pair of limbs (fore limbs or hind limbs) during the stance phase of locomotion.

- Swing time

Swing time refers to the duration of the swing phase in gait analysis, during which the paw is lifted off the ground and moves forward in preparation for the next step.

- Paw Angle

The paw angle refers to the orientation of the paw relative to the direction of forward movement during the stance phase. It is typically measured as the angle between the longitudinal axis of the paw and the body axis or direction of travel.

- Sum Paw Angle

The sum paw angle was calculated by subtracting the paw angle of the left paw (multiplied by −1) from that of the corresponding right paw.

### 2.4. Determination of Body Weight and Brain Weight

The body weights of the mice were measured twice a week throughout the experiment using a precision scale (Kern EMS, Kern & Sohn GmbH, Balingen; Germany). The brains of the mice were weighed immediately after removal on the day of perfusion using a precision scale (Kern EMS, Kern & Sohn GmbH, Balingen; Germany).

### 2.5. Tissue Preparation and Immunohistochemistry

Transcardiac perfusion, brain sampling, and tissue fixation were performed according to protocols described previously [38]. In brief, mice were deeply anesthetized with i.p. ketamine (100 mg·kg^−1^) and xylazine (10 mg·kg^−1^) and transcardially perfused with ice-cold PBS (Phosphate-buffered saline), followed by a cold 3.7% formalin solution (pH 7.4). After overnight post-fixation in the same fixative, the brains were dissected and embedded in paraffin, and coronal 5-μm-thick sections were prepared for immunohistochemistry. The coronal sections were analyzed at level 215 and level 265 according to the mouse brain atlas published by Sidman et al. (http://www.hms.harvard.edu/research/brain/atlas.html (accessed on 21 March 2025)).

The respective immunohistochemical stains of the brain sections of cuprizone-intoxicated mice and control mice were performed on the same days and under the same conditions. For immunohistochemistry, sections were rehydrated and, if necessary, antigens were unmasked with heat in a TRIS/EDTA (pH 9.0) or citrate (pH 6.0) buffer. After washing in PBS, sections were blocked in blocking solution (i.e., serum of the species in which the secondary antibodies were produced) for 1 h. The sections were then incubated overnight (4 °C) in primary antibodies diluted in the blocking solution. Appropriate negative controls (omission of primary antibodies or application of isotype control antibodies) were performed in parallel experiments. The following primary antibody concentrations were applied: anti-IBA1 antibodies (1:5000; Wako; #019-19741; RRID: AB_839504) and anti-PLP antibodies (1:5000, BIO-RAD; #MCA-839G; RRID: AB_2237198). The next day, the slides were treated with 0.35% hydrogen peroxide in PBS for 30 min. After washing in PBS, the slides were incubated in biotinylated secondary antibody solution at room temperature for 1 h. The following secondary antibody concentrations were applied: anti-rabbit secondary antibodies (1:200; Vector; #BA-1000; RRID: AB_2313606) and anti-mouse antibodies (1:200; Vector, #BA-9200, RRID: AB_2336171). Immunohistochemical labeling was visualized using the avidin–biotin–peroxidase complex method (ABC kit; Vector Laboratories, Peterborough, UK) and 3,3′-diaminobenzidine (DAKO, Hamburg, Germany). Sections were finally mounted in DePeX (18,243.02, Serva, Heidelberg, Germany). The slides were scanned and digitized at high resolution using a scanning microscope (Grundium Oy, Tampere, Finland).

### 2.6. Selected Regions of Interest (ROI)

Immunohistochemical stains were performed on two consecutive sections in rostro-caudal sequence each from the region at the level of the commissura anterior (region 215 according to the mouse brain atlas by Sidman et al.) (http://www.hms.harvard.edu/research/brain/atlas.html) and from the level of the rostral hippocampus (region 265 according to the mouse brain atlas by Sidman et al.) [39]. At the level of the commissura anterior, the lateral part of the corpus callosum (latCC), the caudate–putamen complex (CPu, striatum), and the motor cortex (MC) were defined as ROI. At the level of the rostral hippocampus, the medial part of the corpus callosum (medCC) and the CPu were defined as ROI.

To delineate the medCC in region 265 as a ROI and the latCC in region 215 as a ROI, the following procedure was used for the digital images of the brain sections: From the highest point of the CC in the left and right hemispheres, a perpendicular line was drawn through the CC, dividing the CC into a medial portion and two lateral portions. The medial portion was then outlined in region 265 using the polygon tool in Image J (version 1.53k; JAVA 1.8.0_172 [64-bit]; Bethesda, MD, USA). In region 215, the lateral portions of the CC located perpendicular to the perpendicular lines were outlined as ROIs in Image J.

The CPu was clearly visible in all stains on the digital images and was also marked using the polygon tool in ImageJ (version 1.53k; JAVA 1.8.0_172 [64-bit]; Bethesda, MD, USA) both in region 215 as a separate ROI and in region 265 as a separate ROI.

The motor cortex was marked as an ROI in region 215 using the polygon tool in Image J. To do this, an angle bisector was drawn through the most dorsal angle of the cingulum. This angle bisector was extended to the dorsal cerebral cortex. The medial boundary of the motor cortex was set where the extended angle bisector crossed the cerebral cortex. A second straight line was drawn by dropping a perpendicular line from the most lateral angle of the cingulum to the dorsal cortical surface. Where this line intersected the cortex, the lateral boundary of the motor cortex was defined, and the motor cortex was then delineated as an ROI using the polygon tool in Image J.

Planimetric measurements were performed exclusively within the ROIs.

### 2.7. Planimetric Analyses

For the planimetric analyses, stained sections were digitized using a digital scanner (Grundium Oy, Ocus Slide Scanner, Tampere, Finland), including the respective ROIs. Images were subsequently converted into greyscale images in Image J and a threshold was set semi-automatically to filter out unspecific background stains. The images were then converted into binary images and the relative staining intensity of positively stained areas in the respective ROI was calculated. The results of the planimetric analyses are presented as relative staining intensity of the immunoreactive area in relation to the total area of the respective ROI. The planimetric analyses were carried out automatically and with the same settings for all processed sections. The experimenter was, thus, not blinded during the analyses.

### 2.8. Statistics

The statistical analyses were conducted using the software programs Excel (Microsoft, Redmond, WA, USA) and GraphPad Prism (version 8.4.3, GraphPad Software, Boston, MA, USA). To assess the distribution of gait metrics, the Shapiro–Wilk test was used to evaluate normality. If data were normally distributed, a one-way ANOVA was performed to detect significant differences between the groups, followed by Tukey’s multiple comparison test. For non-normally distributed data, the Friedman test was applied, followed by Dunn’s multiple comparisons test. In both post hoc analyses, each time point was compared pairwise with all other time points. The graphs in the results section of the manuscript display only statistically significant differences between the first test day (−1) and all subsequent test days. A comprehensive overview of the full statistical analysis can be found in the Supplementary Materials of this manuscript. The data from the repeated weight measurements of the mice were tested for normal distribution using the Shapiro–Wilk test. Since the data were normally distributed, a one-way ANOVA was performed with a subsequent Tukey’s multiple comparison test. Only statistically significant differences between the weight measurement before the cuprizone intoxication and the measurements during the cuprizone intoxication were indicated and marked with asterisks in the diagram. The comprehensive overview of the complete statistical analysis can be found in the Supplementary Materials to this manuscript (Tables S1–S35).

To compare the data of brain weights, body weights, and staining intensities, non-parametric data were assumed due to the low number of control animals (*n* = 4) included in these analyses. These groups were compared by Mann–Whitney tests. To assess correlations between gait parameters and the results of planimetric histological analyses, Pearson’s correlation was applied to normally distributed data, while Spearman’s rank correlation was used for non-normally distributed data. Confidence intervals were not calculated for the Spearman correlations due to the limited sample size.

## 3. Results

### 3.1. Body Weight Development

**Figure 2:**
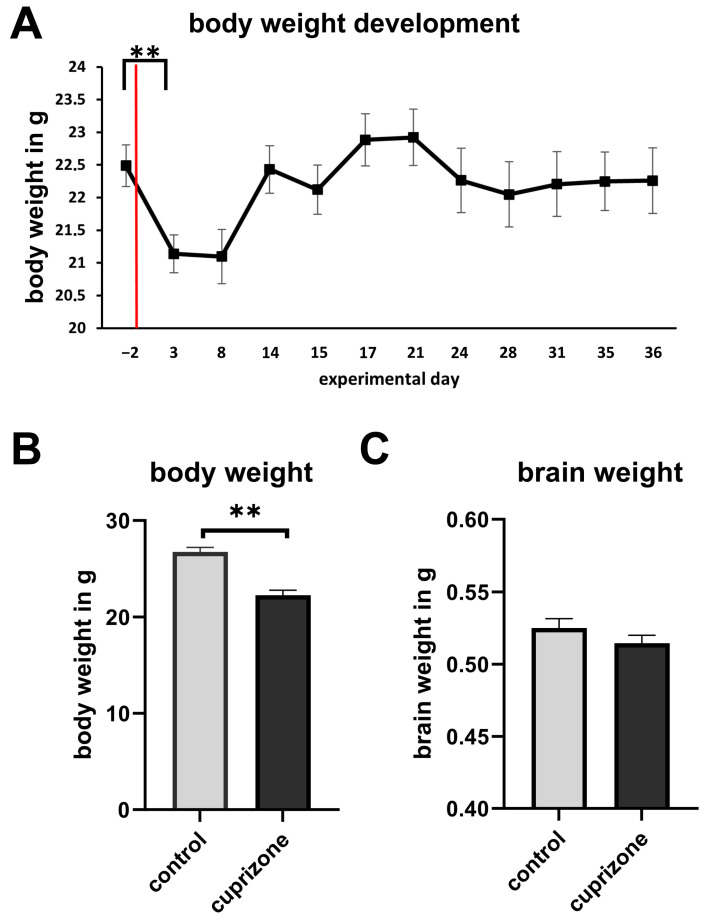
(**A**) Average body weight of mice throughout the study. The red line marks the initiation of cuprizone intoxication. Error bars represent the standard error of the mean (SEM). Statistical analysis was performed using one-way ANOVA followed by Tukey’s post hoc test, comparing pre-intoxication body weights to each subsequent time point. A significant reduction in body weight was observed shortly after the onset of cuprizone treatment. (**B**) Cuprizone-intoxicated mice (*n* = 9) exhibited significantly lower body weights compared to control mice (*n* = 4). Statistical significance was determined using the Mann–Whitney U test. (**C**) Mean brain weights on the day of perfusion. Data are presented as mean ± SEM. ** *p* < 0.01.

The mean body weight of the mice prior to the cuprizone intoxication (day −2) was 22.49 ± 0.336 g (mean ± SEM). This significantly decreased to 21.14 ± 0.306 g three days after the initiation of cuprizone intoxication (*p* = 0.0041; MD: 1.349; 95% CI [0.463 g, 2.234 g] one-way ANOVA followed by Tukey’s multiple comparison test) and further to 21.10 ± 0.441 g by day eight. Over the following 13 days, the body weight gradually increased, reaching a peak average of 22.92 ± 0.459 g. Thereafter, the mean body weight slightly decreased and stabilized at a plateau until the end of the experiment (Figure 2A). On the day of perfusion, a significant difference in mean body weight was observed between cuprizone-intoxicated mice and control mice (*p* < 0.0014; d = −3.094; one-way Mann–Whitney test). Control mice exhibited an average body weight of 26.74 ± 0.46 g, whereas cuprizone-intoxicated mice weighed 22.26 ± 0.53 g. Thus, cuprizone treatment resulted in a significant weight reduction of 4.48 g, corresponding to a 16.75% decrease compared to controls (Figure 2B).

### 3.2. Brain Weight

Immediately after perfusion, brains were removed and weighed. The mean brain weight of control mice was 0.525 ± 0.006 g, compared to 0.514 ± 0.006 g in the cuprizone-intoxicated group. This difference was not statistically significant (*p* = 0.108; d = −0.674; one-tailed Mann–Whitney U test; see Figure 2C).

### 3.3. DigiGait Test—Analysis of Gait Parameters

For the statistical evaluation of the following parameters, measurements from all time points were compared. In this study, just statistical comparisons are shown between baseline values (day –1, prior to cuprizone administration) and all subsequent measurement days during the intoxication period. *p*-values for all other pairwise comparisons are provided in the Supplementary Materials (Tables S1–S35). One mouse from the cuprizone-intoxicated group did not run on the DigiGait apparatus before the start of intoxication and was therefore excluded from further testing and subsequent analyses.

#### 3.3.1. Stride Time

**Figure 3:**
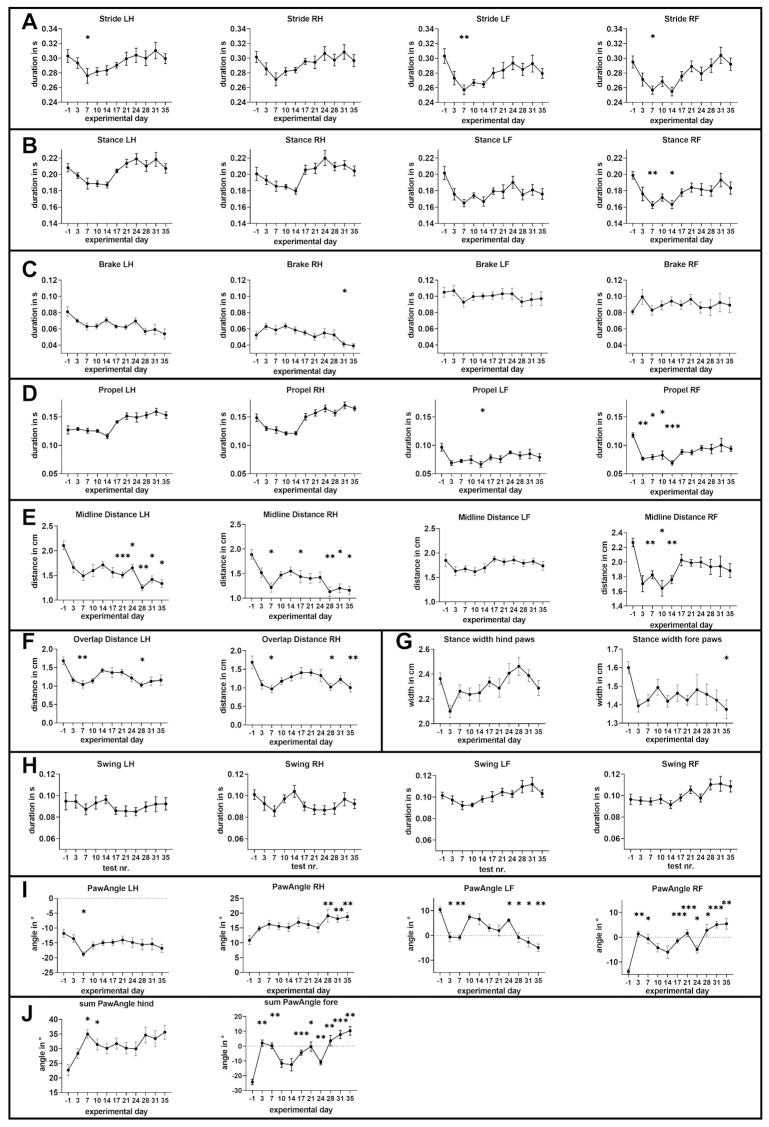
Effects of five weeks of cuprizone intoxication on gait parameters. LH: left hind paw; RH: right hind paw; LF: left fore paw; RF: right fore paw. Asterisks indicate significant differences compared to pre-cuprizone values. Statistical comparisons were conducted between baseline (before cuprizone administration) and all subsequent time points. (**A**) Stride time duration: LH, RH, and RF were analyzed using one-way ANOVA with Tukey’s post hoc test; LF was analyzed using the Friedman test followed by Dunn’s post hoc test. (**B**) Stance phase duration: all paws analyzed using one-way ANOVA with Tukey’s post hoc test. (**C**) Brake phase duration: RH, RF, and LF analyzed with one-way ANOVA and Tukey’s post hoc test; LH analyzed using the Friedman test and Dunn’s post hoc test. (**D**) Propulsion phase duration: RH and LF analyzed with one-way ANOVA and Tukey’s post hoc test; LH and RF analyzed using the Friedman test and Dunn’s post hoc test. (**E**) Midline distance: RH, LH, and RF analyzed using one-way ANOVA with Tukey’s post hoc test; LF analyzed using the Friedman test and Dunn’s post hoc test. (**F**) Overlap distance: analyzed using the Friedman test, followed by Dunn’s post hoc test. (**G**) Stance width: hind paws analyzed with one-way ANOVA and Tukey’s post hoc test; fore paws analyzed using the Friedman test and Dunn’s post hoc test. (**H**) Swing time: RF analyzed using one-way ANOVA and Tukey’s post hoc test; RH, LH, and LF analyzed using the Friedman test and Dunn’s post hoc test. (**I**) Paw angle: LH, LF, and RF analyzed using one-way ANOVA with Tukey’s post hoc test; RH analyzed using the Friedman test and Dunn’s post hoc test. The dashed line marks 0°. (**J**) Sum of paw angles: all values analyzed using one-way ANOVA with Tukey’s post hoc test. The dashed line marks 0°. Data are presented as mean ± SEM (*n* = 8). * *p* ≤ 0.05, ** *p* ≤ 0.01, *** *p* ≤ 0.001.

In gait analysis, stride time refers to the time taken to complete a full gait cycle for one leg, from the initial contact of one paw to the next initial contact of the same paw. It is a key temporal parameter used to evaluate walking patterns and assess locomotor function. As shown in Figure 3A, within one week of cuprizone intoxication, a significant shortening of stride time was observed. For the left hind paw, the stride time decreased from 0.303 ± 0.009 s to 0.276 ± 0.01 s (*p* = 0.05; MD: 0.027 s; 95% CI [0.00004 s, 0.05408 s] ANOVA with Tukey post hoc test). Similarly, the left front paw showed a reduction from 0.303 ± 0.01 s to 0.257 ± 0.007 s (*p* = 0.003; Friedman test with Dunn’s multiple comparison test), and the right front paw dropped from 0.295 ± 0.008 s to 0.257 ± 0.006 s (*p* = 0.032; MD: 0.0383 s; 95% CI [ 0.0033 s, 0.0731 s]; ANOVA with Tukey post hoc test). A similar trend was noted for the right hind paw. Over the subsequent three weeks, stride time gradually returned to nearly its initial values, indicating recovery.

#### 3.3.2. Stance Phase

The stance phase in mouse gait analysis refers to the portion of the locomotion cycle during which the paw is in contact with the ground, supporting the animal’s body weight. It begins when the paw touches the ground (paw strike) and ends when the paw lifts off (paw-off). As shown in Figure 3B, following cuprizone intoxication, changes in the stance phase duration showed a similar pattern for all paws with an initial decline and partial (fore paws) or complete (hind paws) recovery. The initial decline was significant for the right fore paw (from 0.1988 ± 0.004 s to 0.1624 ± 0.004 s; *p* = 0.0032; MD: 0.0363 s; 95% CI [0.0142 s, 0.0584 s]; ANOVA with Tukey post hoc test), while only a trend was observed for the other paws (left fore paw *p* = 0.0552; right hind paw *p* = 0.3953 and left hind paw *p* = 0.1323; ANOVA with Tukey post hoc test for all).

#### 3.3.3. Brake Phase

The brake phase refers to the portion of the stance phase where the paw applies decelerating forces against the ground, typically to reduce forward momentum or control movement. It occurs immediately after paw contact and precedes the subsequent propulsion phase. As shown in Figure 3C, the duration of the brake phase in the right hind paw was significantly reduced on day 31 compared to baseline (from 0.052 ± 0.005 s to 0.041 ± 0.004 s; *p* = 0.047; MD: 0.0111 s; 95% CI [0.0002 s, 0.0221 s]; one-way ANOVA with Tukey’s post hoc test). Notably, we also observed side-specific differences, with the brake phase duration being consistently longer in the left hind paw compared to the right.

#### 3.3.4. Propel Phase

The propel phase is the portion of the stance phase where the paw generates accelerating forces against the ground, pushing the body forward. It follows the brake phase and ends with the paw’s lift-off. As shown in Figure 3D, for the fore paws we initially observed a moderate decline in the duration of the propel phase (left fore paw from 0.097 ± 0.007 s at day −1 to 0.067 ± 0.006 s until day 14 with *p* = 0.031; MD: 0.0303 s; 95% CI [0.0028 s, 0.0577 s]; ANOVA with Tukey post hoc test; right fore paw from 0.118 ± 0.004 s at day −1 to 0.069 ± 0.004 s until day 14 with *p* < 0.0001; Friedman test with Dunn’s multiple comparison) which did, however, not recover.

#### 3.3.5. Midline Distance

The midline distance refers to the distance between the center of each of the paws at maximum ground contact during a stride and the median horizontal plane of the animal’s body. As shown in Figure 3E, for both hind paws we observed a fast and significant drop of the midline distance which was, for the left hind paw the first time significant at day 21 (from 2.108 ± 0.089 cm to 1.508 ± 0.059 cm with *p* = 0.001; MD: 0.6 cm; 95% CI [0.3052 cm to 0.8948 cm]; ANOVA with Tukey post hoc test), and for the right hind paw at day 7 (from 1.889 ± 0.099 cm to 1.216 ± 0.096 cm with *p* = 0.013; MD: 0.67 cm; 95% CI [0.1530 cm to 1.192 cm]; ANOVA with Tukey post hoc test). Furthermore, we observed a reduction in the midline distance in the fore paws; however, this reduction was less pronounced and only significant for the right fore paw (right fore paw from 2.266 ± 0.058 cm to 1.825 ± 0.054 cm until day 7 with *p* = 0.002; MD: −0.4413 cm; 95% CI [−0.6918 cm to −0.1907 cm]; ANOVA with Tukey post hoc test).

#### 3.3.6. Overlap Distance

Overlap distance refers to the horizontal distance between the placement of the fore paw and the hind paw on the same side during a step cycle. As shown in Figure 3F, both hind paws exhibited an initial significant decline in overlap distance, followed by a modest recovery, and subsequently a further decline. The most pronounced decline of the overlap distance for the left hind limb was measured at day 28 from 1.684 ± 0.103 cm to 1.029 ± 0.061 cm with *p* = 0.022; Friedman test with Dunn’s multiple comparison) and for the right hind limb the maximum decline was measured at day 7 (from 1.689 ± 0.168 cm (day −1) to 0.974 ± 0.1 cm with *p* = 0.011; Friedman test with Dunn’s multiple comparison).

#### 3.3.7. Stance Width

Stance width refers to the vertical distance between the paws of the same pair of limbs (fore limbs or hind limbs) during the stance phase of locomotion. As shown in Figure 3G, the hind paws displayed an initial non-significant decline in stance width, followed by a modest recovery and a subsequent further decline. Similarly, the fore paws also showed an initial decline in stance width, followed by a modest recovery and a further decline. The reductions in stance width for the fore paws were significant (from 1.600 ± 0.033 cm to 1.375 ± 0.051 cm by the end of the experiment, with *p* = 0.029; Friedman test with Dunn’s multiple comparison).

#### 3.3.8. Swing Time

Swing time refers to the duration of the swing phase in gait analysis, during which the paw is lifted off the ground and moves forward in preparation for the next step. As demonstrated in Figure 3H, no significant changes were observed in either paw at any time point.

#### 3.3.9. Paw Angle

The paw angle refers to the orientation of the paw relative to the direction of forward movement during the stance phase. It is typically measured as the angle between the longitudinal axis of the paw and the body axis or direction of travel. For the left paws, outward angles are assigned negative values, while for the right paws, outward angles are assigned positive values. If the paws turn inward—meaning the toes are closer to the median sagittal plane than the heel—the angles are positive for the left paws and negative for the right paws. As demonstrated in Figure 3I, at the beginning of the experiment, the hind paws pointed outward, and this gradually increased throughout the cuprizone intoxication. For the left hind paw (from −11.81 ± 1.278° to −18.79 ± 0.776° until day 7 with *p* = 0.048; MD: 6.981°; 95% CI [ 0.0562°, 13.91°]; ANOVA with Tukey post hoc test) and the right hind paw (from 10.84 ± 1.52° to 19.08 ± 2.19° until day 28 with *p* = 0.005; Friedman test with Dunn’s multiple comparison), the increase in the outward-orientated paw angle was significant. In contrast, at the beginning of the experiment, both fore paws pointed inward. During cuprizone-induced demyelination, the inward-orientated paw angle decreased at both sites. At the end of the experiment, both fore paws pointed outward (left fore paw from 10.46 ± 1.104°to −4.906 ± 1.497° until day 35 with *p* = 0.002; MD: 15.37°; 95% CI [6.853°, 24.15°]; right fore paw from −13.78 ± 0.928° to 5.456 ± 2.146° until day 35 with *p* = 0.003; MD: 19.23°; 95% CI [−30.61°, −7.855°]; ANOVA with Tukey post hoc test).

#### 3.3.10. Sum Paw Angle

The sum paw angle was calculated by subtracting the paw angle of the left paw (multiplied by −1) from that of the corresponding right paw. For the hind paws, this value was initially 22.65 ± 1.93° and increased significantly to 35.04 ± 1.45° one week after the onset of cuprizone intoxication (*p* = 0.036; MD: 12.39°; 95% CI [0.8947°, 23.88°] one-way ANOVA with Tukey’s post hoc test), indicating a progressive abduction of the hind limbs. In contrast, the fore paws initially exhibited a pronounced adducted position, reflected by a negative sum paw angle of −24.24 ± 1.90°. By the end of the experiment, this value had increased significantly to 10.36 ± 2.99° (*p* = 0.001; MD: 34.6°; 95% CI [16.62°, 52.58°]; one-way ANOVA with Tukey’s post hoc test), indicating a shift toward abduction in the forelimbs.

### 3.4. Histology and Immunohistology

#### 3.4.1. Anti-PLP-Immunohistochemistry and Planimetric Analysis

**Figure 4:**
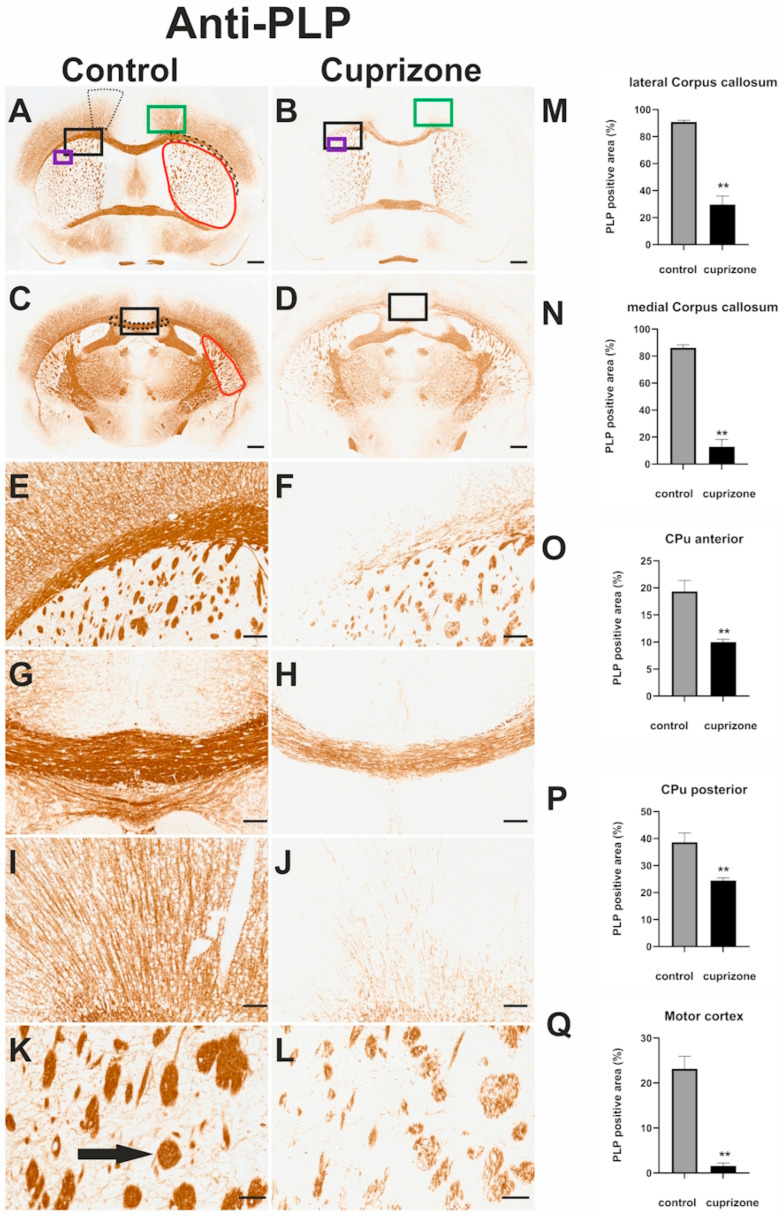
Representative images of anti-PLP immunohistochemistry (Myelination, **A**–**L**) processed sections and planimetric analyses of brain sections stained immunohistochemically for PLP (**M**–**Q**). Frontal sections through the telencephalon of a control mouse (**A**,**C**,**E**,**G**,**I**,**K**) and a cuprizone-intoxicated mouse (**B**,**D**,**F**,**H**,**J**,**L**) immunohistochemically stained for PLP. The sections were made at the level of the Commissura anterior ((**A**,**B**); region 215 according to the mouse brain atlas by Sidman et al.) and at the level of the rostral part of the hippocampus ((**C**,**D**); region 265 according to the mouse brain atlas by Sidman et al.). The area outlined in dots in (**A**) represents an example ROI for the MC. The area outlined in dashed lines in (**A**) represents an example ROI for the latCC. The area outlined in red in (**A**,**C**) represents an example of an ROI for the CPu. The area outlined in dotted lines in (**C**) represents an example of the ROI for the medCC. (**E**,**F**) Higher magnifications of the lateral CC. (**E**,**F**) correspond to the sections marked with the large black rectangle on the left in (**A**,**B**). In (**F**), a clear reduction in myelination of the lateral CC can be seen in comparison with E. (**G**,**H**) Higher magnifications of the medial CC marked with a rectangle in (**C**,**D**). A clear reduction in myelination of the lateral CC can also be seen in (**H**) compared with (**G**). (**I**,**J**) Enlarged details of the MC, marked in (**A**,**B**) with the green rectangles. The fibers in the MC of cuprizone-intoxicated mice (**J**) exhibit significantly weaker myelination than in the MC of control animals (**I**). (**K**,**L**) show enlarged sections of the CPu, which are marked in (**A**,**B**) by small purple rectangles. Corticostriatal and corticospinal fiber bundles can be seen here. These are less myelinated in cuprizone-intoxicated animals (**L**) than in control animals (**K**). The arrow in (**K**) marks an example of a fiber tract running through the CPu, in this case corticostriatal and/ or corticospinal fibers. The scale bar in (**A**) corresponds to 500 µm and is valid for (**A**–**C**) and (**D**). The scale bar in (**E**) corresponds to 100 µm and is valid for (**E**–**I**) and (**J**). The scale bar in K corresponds to 50 µm and is valid for K and L. (**M**–**P**) Relative staining intensity of PLP-positive areas in sections of the lateral CC ((**M**); (region 215 according to the mouse brain atlas by Sidman et al.), the medial CC ((**N**); (region 265 according to the mouse brain atlas by Sidman et al.), the anterior CPu (**O**) (region 215 according to the mouse brain atlas by Sidman et al.), the posterior CPu (**P**) (region 265 according to the mouse brain atlas by Sidman et al.), and the MC (**Q**). For semi-automatic detection of these immunoreactive areas, the experimenter selected a threshold in Image J that covers a maximum of specifically immunohistochemically labeled structures in all preparations from all animals and does not detect non-specific background stains. The black bars represent mean values obtained from brains of cuprizone-intoxicated mice (*n* = 8) and the light gray bars represent mean values obtained in untreated control mice (*n* = 4). The error bars always represent SEM. *p*-values: ** ≤ 0.01.

To confirm that the observed gait alterations were associated with CNS demyelination, we performed PLP immunohistochemistry and analyzed motor-related brain regions. Cuprizone-intoxicated mice exhibited a marked reduction in PLP immunoreactivity compared to controls, particularly in the CC, MC, and corticostriatal fibers of the CPu (Figure 4).

In the CC, the relative staining intensity of PLP-positive areas in the lateral CC decreased from 90.9 ± 1.13% in controls to 29.73 ± 6.22% in cuprizone-treated mice (*p* = 0.002; *d* = −4.144; Mann–Whitney test), and in the medial CC from 86.11 ± 2.23% to 12.78 ± 5.45% (*p* = 0.002; *d* = −5.589; Mann–Whitney test), (Figure 4M,N).

In the CPu, the anterior region showed a reduction from 19.34 ± 2.03% in controls to 9.98 ± 0.57% in cuprizone-treated mice (*p* = 0.002; d = −3.603; Mann–Whitney test), and the posterior region from 38.63 ± 3.43% to 24.46 ± 0.99% (*p* = 0.002; *d*= −1.293; Mann–Whitney test), (Figure 4O,P).

In the MC, the relative staining intensity of PLP-positive areas was significantly reduced from 23.16 ± 2.77% in control mice to 1.59 ± 0.66% following cuprizone treatment (*p* = 0.002; *d* = −6.319; Mann–Whitney test), (Figure 4Q).

These findings confirm extensive demyelination in key motor-associated CNS regions following cuprizone exposure.

#### 3.4.2. Anti-IBA1-Immunohistochemistry and Planimetric Analysis

**Figure 5:**
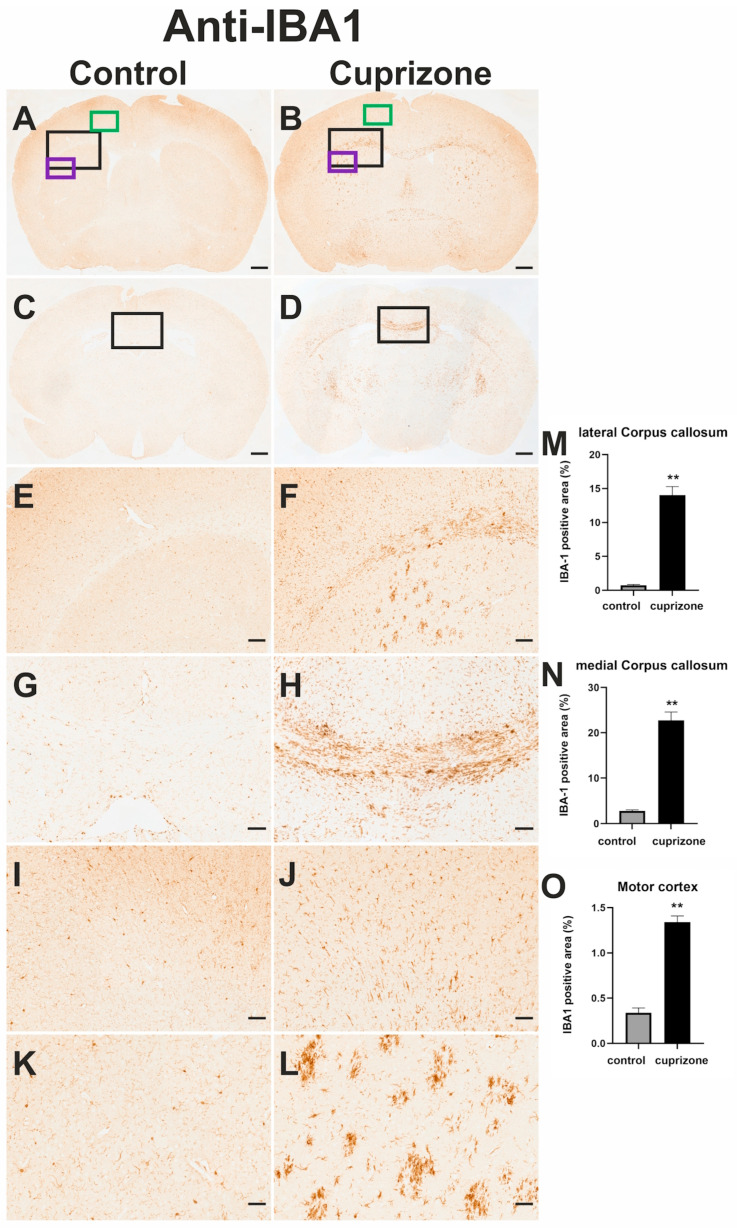
Immunohistochemistry for microglia (IBA1, **A**–**L**) and planimetric analysis of brain sections stained immunohistochemically for IBA1. Frontal sections through the telencephalon of a control mouse (**A**,**C**,**E**,**G**,**I**,**K**) and a cuprizone-intoxicated mouse (**B**,**D**,**F**,**H**,**J**,**L**) immunohistochemically stained for IBA1. The sections were made at the level of the Commissura anterior (**A**,**B**; region 215 according to the mouse brain atlas by Sidman et al.) and at the level of the rostral part of the hippocampus ((**C**,**D**); (region 265 according to the mouse brain atlas by Sidman et al.). (**E**,**F**) Higher magnifications of the lateral CC. (**E**,**F**) correspond to the sections marked with the large black rectangles in (**A**,**B**). In (**F**), an enhanced immunoreactivity for IBA1 can be seen in comparison with (**E**). (**G**,**H**) Higher magnifications of the medial CC marked with a black rectangle in (**C**) and (**D**). In (**H**) compared to (**G**), an increased immunoreactivity for IBA1 in the medial CC can be recognized. (**I**,**J**) Enlarged sections of the MC, marked in (**A**,**B**) with the green rectangles. The anti-IBA1 immunoreactivity is also increased in (**J**) compared to (**I**). (**K**,**L**) show enlarged sections of the CPu, which have been marked in (**A**,**B**) with the purple rectangles. The corticostriatal fiber tracts of the cuprizone-intoxicated animal (**L**) show increased anti-IBA1 immunoreactivity compared to the control animal (**K**). The scale bars in (**A**) correspond to 500 µm and are valid for (**A**–**D**). The scale bar in (**E**) corresponds to 100 µm and is valid for (**E,F**). The scale bar in (**G**) corresponds to 100 µm and is valid to (**G**,**H**). The scale bar in (**I**) corresponds to 50 µm and is valid to (**I**–**L**). (**M**−**O**): Relative staining intensity of IBA1-positive areas in sections of the lateral CC (**M**), (region 215 according to the mouse brain atlas by Sidman et al.), the medial CC (**N**), (region 265 according to the mouse brain atlas by Sidman et al.), and the MC (**O**). The staining intensity is expressed as a percentage of the area of an ROI that was specifically immunoreactive. The black bars represent mean values obtained in brains of cuprizone-intoxicated mice (*n* = 8) and the light gray bars represent mean values obtained in untreated control mice (*n* = 4). The error bars always represent SEM. *p*-values: ** ≤ 0.01.

A significant increase in immunoreactivity for IBA1 was observed in cuprizone-intoxicated mice compared to control mice in the CC, MC, and fiber tracts of the CPu (Figure 5A–L). In contrast to control mice, the microglial cells of cuprizone-intoxicated mice showed shorter processes and a more amoeboid morphology (see Supplementary Figure S1). In cuprizone-intoxicated mice, clear signs of inflammatory processes were detected in various areas of the brain. The proportion of IBA1-positive areas in the medial CC, lateral CC, and MC was significantly higher than in control animals (Figure 5M–O).

##### Corpus Callosum (CC)

The relative staining intensity of IBA1-positive area was 0.742 ± 0.122% in the lateral CC (region 215 according to the mouse brain atlas by Sidman et al.) of control mice and 14.03 ± 1.262% in the lateral CC of cuprizone-intoxicated mice. The Mann–Whitney test showed that this difference was highly significant (*p* < 0.0014; *d* = 4.113; Figure 5M). In the medial CC (region 265 according to the mouse brain atlas by Sidman et al.) the relative staining intensity of IBA1 positive area was 2.82 ± 0.189% in control mice and 22.75 ± 1.805% in cuprizone-intoxicated mice (Mann–Whitney test, *p* = 0.0014; *d* = 4.314; Figure 5N).

##### Motor Cortex

In the MC, the relative staining intensity of IBA1-positive area was 0.34 ± 0.052% in control mice and 1.339 ± 0.069% in cuprizone-intoxicated mice (Mann–Whitney test, *p* = 0.0014; *d* = 5.437; Figure 5O).

### 3.5. Correlation of Gait Parameters with Histological Findings

Next, we were interested in whether the extent of pathological changes, as detected by immunohistochemistry, correlates with the extent of gait abnormalities. To this end, Pearson’s correlation was applied to normally distributed data, while Spearman’s rank correlation was used for non-normally distributed data. Of note, significant correlations between several gait parameters and the degree of myelination of the CC were detected.

#### 3.5.1. Correlation of the Relative Staining Intensity of PLP-Positive Areas in the Anterior Lateral CC with Gait Parameters

**Figure 6:**
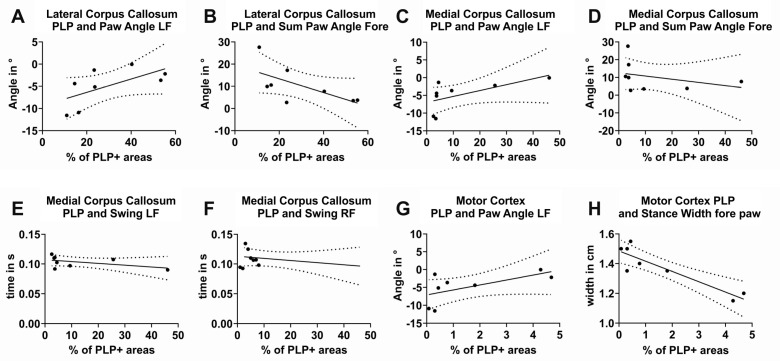
Regressions of relative PLP staining intensities and values of specific gait parameters. (**A**–**H**): Scatter plots of the parameters measured in DigiGait that correlate significantly with the PLP-immunoreactive parts of the lateral MC, the medial CC, or the MC. Regression lines are displayed as solid lines and confidence intervals as dashed lines. Relative staining intensity in the lateral CC (region 215 according to the mouse brain atlas by Sidman et al.), and (**A**) paw angle of the left fore paw and (**B**) sum paw angle of the fore paws. Relative staining intensity in the medial CC (region 265 according to the mouse brain atlas by Sidman et al.) and (**C**) paw angle of the left fore paw, (**D**) sum paw angle of the fore paws, (**E**) swing time of the left fore paws, and (**F**) swing time of the right fore paws. Relative staining intensity in MC and (**G**) paw angle of the left hind paw and (**H**) stand width of the fore paws.

The relationship between myelination in the latCC and gait parameters was assessed using Pearson’s correlation analysis (Figure 6). Significant correlations were observed between the relative staining intensity of PLP-positive area in the lateral CC and specific gait features. A positive correlation was found with the paw angle of the left fore paw (*r* = 0.626, *p* = 0.0485; 95% CI: –0.141 to 0.923; Figure 6A), while a negative correlation emerged with the combined paw angle of both fore paws (*r* = –0.645, *p* = 0.0422; 95% CI: –0.928 to 0.11; Figure 6B). These findings suggest that the extent of callosal myelination influences forelimb coordination during gait.

#### 3.5.2. Correlation of the Relative Staining Intensity of PLP-Positive Areas in the Posterior Medial CC with Gait Parameters

Spearman’s rank-order analysis revealed significant correlations between the extent of PLP immunoreactivity in the medCC and several gait parameters (Figure 6). A strong positive correlation was observed with the paw angle of the left fore paw (*ρ* = 0.8982, *p* = 0.0023; Figure 6C). In contrast, PLP-positive area in the medial CC showed negative correlations with the combined fore paw angle (*ρ* = –0.6707, *p* = 0.0378; Figure 6D), as well as with the swing time of both the left (*ρ* = –0.7066, *p* = 0.0289; Figure 6E) and right fore paw (*ρ* = –0.6946, *p* = 0.0315; Figure 6F). Confidence intervals could not be determined due to limited sample size.

#### 3.5.3. Correlation of the Relative Staining Intensity of PLP-Positive Areas in the MC with Gait Parameters

Spearman’s rank-order analysis revealed a positive correlation between the relative staining intensity of the PLP-positive area in the MC and the paw angle of the left fore paw (*ρ* = 0.6667, *p* = 0.0415; Figure 6G). Additionally, a negative correlation was observed between MC myelination and the stance width of the fore paws (*ρ* = –0.6747, *p* = 0.0369; Figure 6H). Confidence intervals were not calculated due to the limited sample size.

## 4. Discussion

### 4.1. Successful Induction of Demyelination by Cuprizone Intoxication

The induction demyelination in the CC, the MC, and the CPu by feeding cuprizone was successful. This was directly demonstrated by the qualitative observation and quantitative evaluation of the PLP staining. The initial loss of body weight is also a typical and repeatedly documented phenomenon of successful cuprizone intoxication [35,40]. It was therefore possible to ensure that the animals had ingested sufficient cuprizone via the diet to induce demyelination.

### 4.2. Brain Weight and Ratio Brain Weight/Body Weight

The brain weight was determined because a possible loss of brain weight was used as an indicator of systemic brain atrophy. The difference in mean brain weight between cuprizone-intoxicated and control animals was not significant. This suggests that the demyelination induced by cuprizone is limited to some fiber tracts and is not accompanied by a major loss of brain parenchyma. On the other hand, it should be considered that demyelination can be compensated by reactive gliosis and that a possible loss of mass may be masked [41]. Furthermore, it is known that demyelination in the CNS caused by cuprizone intoxication is usually accompanied by the formation of edema in the CNS [42,43]. A possible increase in brain mass due to edema formation could also mask a possible loss of brain tissue.

### 4.3. Gait

The DigiGait analysis software calculates 39 gait parameters for the fore paws and 43 for the hind paws of each animal. This paper does not discuss all parameters in detail because some of these values are redundant. For example, some parameters are quotients of two other measured values, while others are merely totals of other measured values. In this study, only those parameters that showed a statistically significant difference from the values measured before cuprizone intoxication are shown in more detail.

The gait pattern of mammals is subject to complex control and modeling by the pyramidal and extrapyramidal systems and the cerebellum. It should be mentioned that these systems do not represent real functional entities but are permanently intertwined. In our study, we have demonstrated demyelination and inflammatory reactions in the CC as well as in the CPu.

The motor pathways are crossed (pyramidal tract/corticospinal tract). Therefore, an effective exchange of information between the cerebral hemispheres is important for coordinated gait and coordinated activation of limb and trunk muscles. The CC, which connects the two hemispheres of the telencephalon, plays a prominent role in the sensorimotor coordination of the two halves of the body. The so-called motor CC is known to functionally connect the right and left primary MC [44,45,46]. It has already been described in the scientific literature that lesions or atrophies of the CC are associated with gait disorders in humans [47]. However, Moretti et al. [47] did not specify which specific limitations in particular correspond to damage to the CC. However, it remains undisputed that the degree of lesion load in the CC correlates with the extent of gait disturbance in MS patients [48]. Compliant with this, it has been shown that good bilateral connectivity of both motor cortices can help to functionally compensate for unilateral motor circuit damage [49].

In addition to the CC, both in MS and cuprizone intoxication, more fiber tracts in the CNS are affected by demyelination. For example, we observed demyelination in the fiber tracts that cross or innervate and/or leave the CPu (Figure 4K,L). These are fibers of the inner capsule, corticostriatal fibers, and several other small fiber systems [50]. The inner capsule plays a prominent role in gait, because it is one level of the pyramidal system [51].

### 4.4. Stride Time

The stride time (stride duration) refers to the time of an entire stride cycle for one paw and thus includes both the stance phase and the swing phase. We initially measured a slight shortening of the stride time. Essentially, the stride time depends on the running speed and the cadence of the steps [52]. Since the speed of the treadmill was constant at 15 cm/s during the experiment, the stride frequency (stride cadence) must have increased after the application of cuprizone. That was indeed the case. The data was not shown here due to redundancy. It has been described that an increase in step frequency is associated with so-called “freezing” in Parkinson’s disease [53]. We were able to observe changes in the CPu, which in turn is innervated dopaminergically by the substantia nigra in which dopaminergic neurons are lost in Parkinson’s disease. A decrease in stride time (stride duration) was also reported by Lambert et al. (2014) [54] in an olivocerebellar ataxia model.

### 4.5. Stance Phase

The stance phase is also known as stance duration and includes the entire period during which a paw has contact with the ground in the course of one step. The phase consists of the brake phase and the propel phase. We measured a shortening of the stance phase after cuprizone intoxication. However, the shortening was only significant for the right fore paw. Normally, the stance phase is shortened by increasing the running speed, but in our experiment, the treadmill was always operated at a constant speed of 15 cm/s. Lambert et al. (2014) [54] reported a decrease in stance duration in an olivocerebellar lesion model. A shortening of the stance phase (stance duration) was also observed in a mouse stroke model [55].

It is generally described that in hind limbs, the stance phase can be divided into approximately 2/3rds propel phase and 1/3rd brake phase. We also determined this ratio for the hind paws of the mice investigated. For the front paws, we determined a ratio of propel-phase-to-brake-phase of approximately 50:50. This is also described in the literature [37,56].

The different distribution of brake phase and propel phase within the stride time is also due to the fact that the front paws and hind paws fulfill different tasks in the gait of mice. Thus, the hind paws are more involved in propulsion, and the fore paws are more likely to mediate braking in the course of a stride [57].

We have shown that the stance phase correlates negatively with the degree of myelination of the MC, at least for the fore paws. This result contrasts with the assumption that a shortening of the stance phase is a pathological phenomenon, because a lower degree of myelination in the MC is not automatically associated with a shorter stance phase. It is therefore possible that the reduction in the stance phase is due to a training effect caused by repeated testing on the DigiGait or is associated with the advanced age of the animals. This hypothesis can be tested in future follow-up experiments in which the gait parameters of healthy mice are measured over a period of time on the DigiGait.

### 4.6. Brake Phase

The brake phase is also known as brake time and indicates the duration between the initial paw contact and the maximum paw contact with the ground [37]. Throughout cuprizone intoxication, we measured significant shortening of the brake phase of the hind paws. The braking during the gait is not a simple dropping of the paws but is a complex process in which the paw first hits the ground with the calcaneus and only then is it rolled down to the phalanges. This requires, above all, a coordinated tensing of the extensors on the hock joint when the paw is placed on the ground, followed by a gradual release of this tension. This complex interplay of muscle groups and the gradual release of tension in the extensors can be disturbed by a disorder of the motor circuitry. For example, a shortening of the brake phase (brake duration) was measured in a mouse stroke model [55].

### 4.7. Propel Phase

The propel phase (propulsion duration) is also known as propel time, propulsion, or propulsion time. It indicates the time between the maximum contact of a paw with the ground and the complete detachment of the paw from the ground [37]. The developments after the start of cuprizone intoxication were different for the front and hind paws. While the propel phase shortened in the fore paws, the propel phase tended to lengthen in the hind paws. A decrease in propulsion duration was also reported by Lambert et al. 2014 [54] after the olivocerebellar system had been lesioned.

### 4.8. Midline Distance

The midline is the distance between the center of the individual paws during their maximum contact with the ground in the course of a stride and the median horizontal plane of the animal’s body. An increase in the amount of the midline distance is interpreted as a sign of increased extension of the limbs [58]. However, we detected a reduction in the amount of midline distance for both the hind paws and the front paws after cuprizone intoxication. This could either be due to a reduced extension of the limbs or a bulging of the mice’s torso.

### 4.9. Overlap Distance

The overlap distance indicates the distance between the centers of a fore paw print and the ipsilateral hind paw print. Firstly, the position of the fore paw is determined at a stride, and afterwards the position of the ipsilateral hind paw for a second stride is established, whereafter the distance between the two prints is calculated. The overlap distance shortened significantly during cuprizone intoxication. The shortening of the overlap distance is consistent with the results of the analysis of the midline distance, which was also shortened for all paws after cuprizone intoxication. As a result, the front and hind paw prints must be brought closer together. This may also be due to a reduced extension of the entire limb or an increased arching of the trunk.

### 4.10. Paw Angle and Sum Paw Angle

The paw angle is the angle that the respective paw makes to the longitudinal body axis of the animal. The paw angle is also known as the toe-out angle. All paws were turned significantly further outward throughout the cuprizone intoxication. In our measurements, the paw angle of the left fore paw correlated positively with the degree of myelination of the CC and the MC, whereby the paws were directed further outwards with less myelination. Consistent with the results of the paw angle, the angles between the front paws and between the hind paws, which were measured in the form of the sum paw angle, also increased significantly during the cuprizone intoxication. Our findings are consistent with reports that demyelination in the CNS is associated with an increase in the amount of the paw angle [59]. The enlarged or more outwardly orientated paw angle may represent a compensatory reaction that is to counteract gait instability [60,61,62]. An enlargement of the paw angle was also measured in the olivocerebellar lesion model [54].

### 4.11. Stance Width

The stance width indicates the width between the front paws and the width between the hind paws. We measured a reduction in stance width after cuprizone intoxication. These changes were significant for the front paws, but we initially observed such a trend for the hind paws as well. A small stance width is generally associated with a less stable gait. Other studies have measured a reduction in a number of pathological events, for example, in a lesion model of the mouse sciatic nerve [63]. Akula et al. (2020) [64] showed that the stance width decreases with age and is dependent on body length. Guillot et al. (2008) [65] were able to show that the stance width decreases in a mouse model of amyotrophic lateral sclerosis. And Lambert et al. (2014) [54] showed that the stance width widens in an olivocerebellar lesion model.

### 4.12. Neuroinflammatory Reactions in Demyelinated Structures

Various mechanisms are discussed for the neuroinflammatory reactions underlying demyelination. On the one hand, the immigration of autoimmune-reactive T and B cells into the CNS is considered to be the cause of the inflammatory reaction, on the other hand, it is discussed whether the T and B cells migrate into the CNS only in the course of an already existing inflammation [66,67,68,69].

We observed clear evidence of neuroinflammatory processes in the observed structures. Immunoreactivity for IBA1 was significantly increased in the CC, in the MC, and in the fiber tracts that pass through the CPu. The microglial cells labeled in these structures exhibited an amoeboid morphology with short, plump projections, which is typical for microglial cell activation. It is well known that cuprizone-induced demyelination is also associated with neuroinflammatory processes resulting in microglial cell activation. This result can therefore be seen as confirmation of the successful induction of demyelination in the CC, the MC and the CPu [25,70,71,72,73].

### 4.13. Correlation of Gait Parameters with Histological Measurements

Our results underline that the CC and the myelination of its commissural fibers must influence the gait pattern. The degree of myelination, which we measured using the density of PLP-positive areas, correlates significantly with the paw angle and the swing time of the fore paws.

Myelination within the MC also appears to play a key functional role, as this also correlates with the paw angle and the stance phase of the for paws.

### 4.14. Outlook and Critical Notes

It must be noted that many other areas that are decisive for motor function were not considered in this study, such as the cerebellum or the corticospinal tract.

In the future, further gait analysis methods should be used to investigate the influence of focal demyelination. For example, the proportion of the individual paws in the propulsion and brake forces and their changes should be measured. The so-called CatWalk is suitable for this purpose. The DigiGait apparatus works on the principle of a motor-driven treadmill. The possible artificial influence on some gait parameters due to forced walking should also be ruled out by gait analyses in which the animals voluntarily walk over a surface; the CatWalk is also applicable here. In the CatWalk system, the mice run across a glass plate that is illuminated from the side. The system works differently from the DigiGait apparatus, as it does not use a motor-driven treadmill that forces the mice to run. Instead, after training, the mice run across the glass plate of their own accord and voluntarily. In addition, the device is designed to record the relative pressure exerted by each paw [74,75,76].

The authors are aware that a follow-up experiment should be carried out in the future, in which additional control animals should also be investigated in the DigiGait using the same pattern to investigate the influence of a possible motor learning effect and the impact of aging on the animals.

Several aspects must be considered when translating the present findings to clinical practice. First, the animal model used in this study induces demyelination without the involvement of autoreactive T or B cells. While the contribution of peripheral immune cells diminishes in progressive stages of multiple sclerosis (MS)—both primary and secondary progressive MS—it is well established that these cells play a critical role during the early relapsing–remitting phase. Consequently, the current model may have limited relevance for investigating gait abnormalities in early MS. The histopathological features of relapsing–remitting MS are more accurately recapitulated in the experimental autoimmune encephalomyelitis (EAE) model. Notably, our group has previously demonstrated that specific gait parameters, such as swing time and paw angle, are significantly altered during early EAE development [33].

Second, mice are quadrupeds, whereas humans exhibit bipedal locomotion. These distinct modes of movement impose different biomechanical demands and rely on divergent neural circuits within the pyramidal and extrapyramidal motor systems [77,78,79,80]. Nonetheless, several gait parameters—such as swing phase, stance phase, and stride duration—are conserved and quantifiable across both species [81].

To improve translational validity, future studies should include gait analysis in MS patients with well-characterized lesion topography, as determined by advanced neuroimaging techniques. The use of systems such as the Gait Real-Time Analysis Interactive Lab [82] offers a promising approach. Ultimately, comparative analyses between human gait profiles and those obtained from various demyelination models in rodents may help to identify conserved gait parameters, which could serve as reliable translational markers of motor dysfunction in MS [81]. Particular attention should be paid to the parameter of gait variability in such studies, as it can be recorded in both mice and humans using automated systems such as DigiGait and GRAIL, and pathological changes in the CNS in both species are accompanied by specific changes in gait variability [83].

## 5. Conclusions

The objective of the study was to investigate the effects of cuprizone-induced demyelination on the gait pattern in mice. The successful induction of demyelination in the CNS was demonstrated by qualitative and quantitative histological analyses. The study found that there was no significant difference in brain weight between cuprizone-intoxicated mice and controls, indicating that demyelination was limited to specific fiber tracts and was not associated with a substantial loss of brain parenchyma.

The gait analysis revealed significant alterations in multiple gait parameters, including stride time, stance phase, braking phase, propel phase, midline distance, overlap distance, paw angle, and stance width. These changes were found to be partly associated with the degree of myelination of the CC and MC. Furthermore, the study observed clear evidence of neuroinflammatory processes in the demyelinated structures, including increased immunoreactivity for IBA1 and amoeboid morphology of microglial cells. These results suggest that the CC and the myelination of its commissural fibers play a critical role in gait patterns. The study highlights the importance of further research into the influence of focal demyelination on motor function and the development of new gait analysis techniques to investigate these effects.

## Data Availability

The raw data supporting the conclusions of this article will be made available by the authors on request.

## Supplementary Materials

The following supporting information can be downloaded at: https://www.mdpi.com/article/10.3390/cells14130969/s1, Figure S1: Altered microglial morphology following cuprizone intoxication. Representative IBA1-immunostained microglial cells in coronal brain sections of mice. Images (A) and (B) show cells from the corpus callosum (CC); images (C) and (D) from the motor cortex (MC). In cuprizone-intoxicated animals, microglia exhibit a more amoeboid morphology, with shorter and thicker processes and reduced ramification compared to the highly ramified microglia with long, thin processes observed in controls.; Table S1: RM one-way ANOVA of Body weight; Table S2: Friedman test of Brake LH; Table S3: RM one-way ANOVA of Brake LF; Table S4: RM one-way ANOVA of Brake RF; Table S5: RM one-way ANOVA of Brake RH; Table S6: Friedman test of Midline Distance LF; Table S7: RM one-way ANOVA of Midline Distance LH; Table S8: RM one-way ANOVA of Midline Distance RF; Table S9: RM one-way ANOVA of Midline Distance RH; Table S10: Friedman test of Overlap Distance LH; Table S11: Friedman test of Overlap Distance RH; Table S12: Friedman test of PawAngle RH; Table S13: RM one-way ANOVA of PawAngle LF; Table S14: RM one-way ANOVA of PawAngle LH; Table S15: RM one-way ANOVA of PawAngle RF; Table S16: Friedman test of Propel LH; Table S17: Friedman test of Propel RF; Table S18: RM one-way ANOVA of Propel LF; Table S19: RM one-way ANOVA of Propel RH; Table S20: RM one-way ANOVA of Stance LF; Table S21: RM one-way ANOVA of Stance LH; Table S22: RM one-way ANOVA of Stance RF; Table S23: RM one-way ANOVA of Stance RH; Table S24: Friedman test of StanceWidth LF; Table S25: StanceWidth LH; Table S26: Friedman test of Stride LF; Table S27: RM one-way ANOVA of Stride LH; Table S28: RM one-way ANOVA of Stride RF; Table S29: RM one-way ANOVA of Stride RH; Table S30: RM one-way ANOVA of sum PawAngle fore; Table S31: RM one-way ANOVA of sum PawAngle hind; Table S32: Friedman test of Swing LF; Table S33: Friedman test of Swing LH; Table S34: Friedman test of Swing RH; Table S35: RM one-way ANOVA of Swing RF.
