# Supplementary material for: High-Speed-Ventral-Plane Videography Identifies Specific Gait Pattern Changes in Cuprizone-Induced Demyelination in Mice
Source: Cells. 2025 Jun 24;14(13):969. doi: 10.3390/cells14130969 (PMC12249482; doi:10.3390/cells14130969)
Supplement: Supplementary file 1 [file cells-14-00969-s001.zip › Supplement/Figure S1/Caption Figure S1.docx]

Altered microglial morphology following cuprizone intoxication. Representative IBA1-immunostained microglial cells in coronal brain sections of mice. Images (A) and (B) show cells from the corpus callosum (CC); images (C) and (D) from the motor cortex (MC). In cuprizone-intoxicated animals, microglia exhibit a more amoeboid morphology, with shorter and thicker processes and reduced ramification compared to the highly ramified microglia with long, thin processes observed in controls.
