# Supplementary figures and images for: High-Speed-Ventral-Plane Videography Identifies Specific Gait Pattern Changes in Cuprizone-Induced Demyelination in Mice
Source: Cells. 2025 Jun 24;14(13):969. doi: 10.3390/cells14130969 (PMC12249482; doi:10.3390/cells14130969)

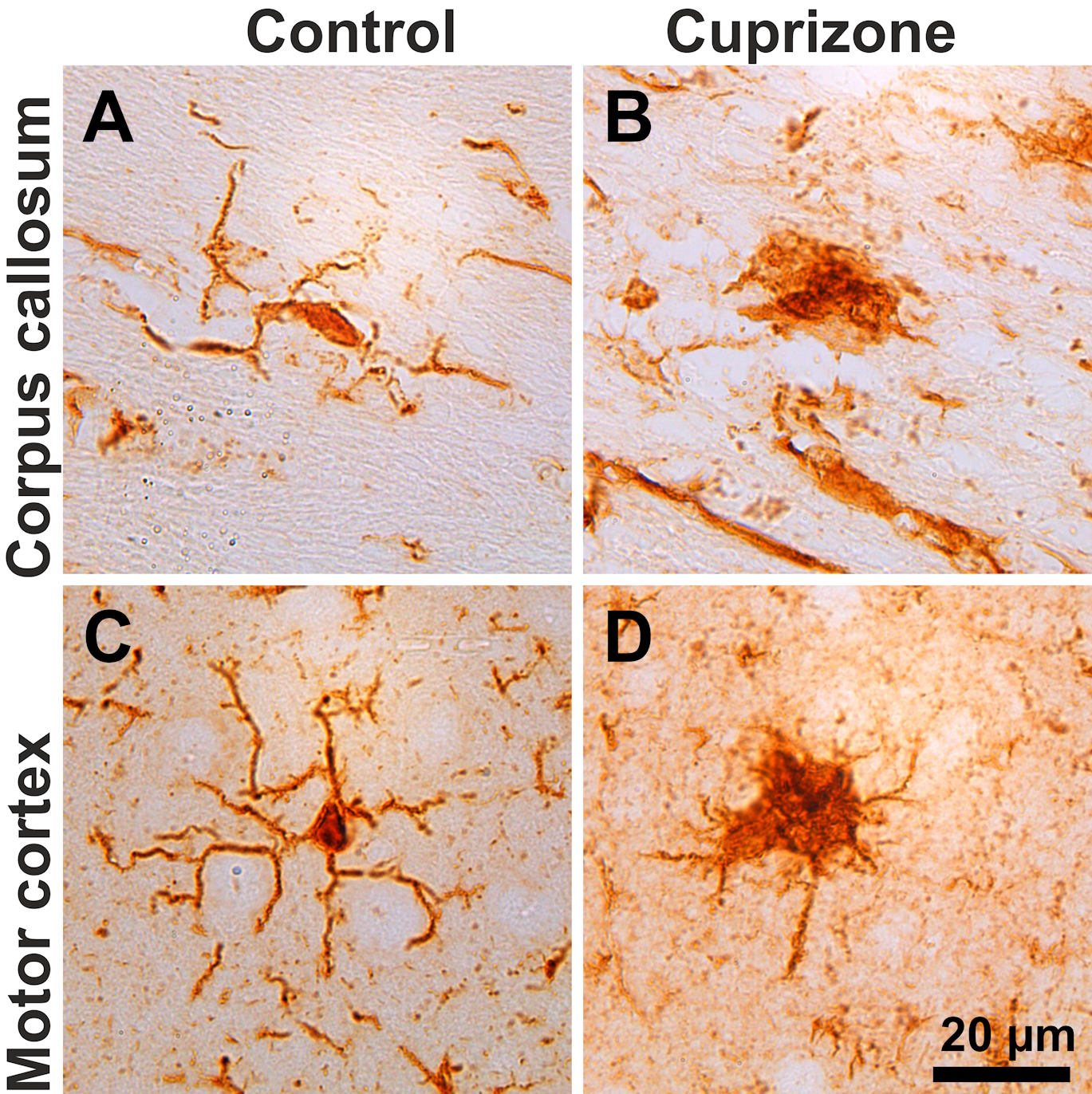

Supplement: Supplementary file 1 [file cells-14-00969-s001.zip › Supplement/Figure S1/Figure S1.jpg]

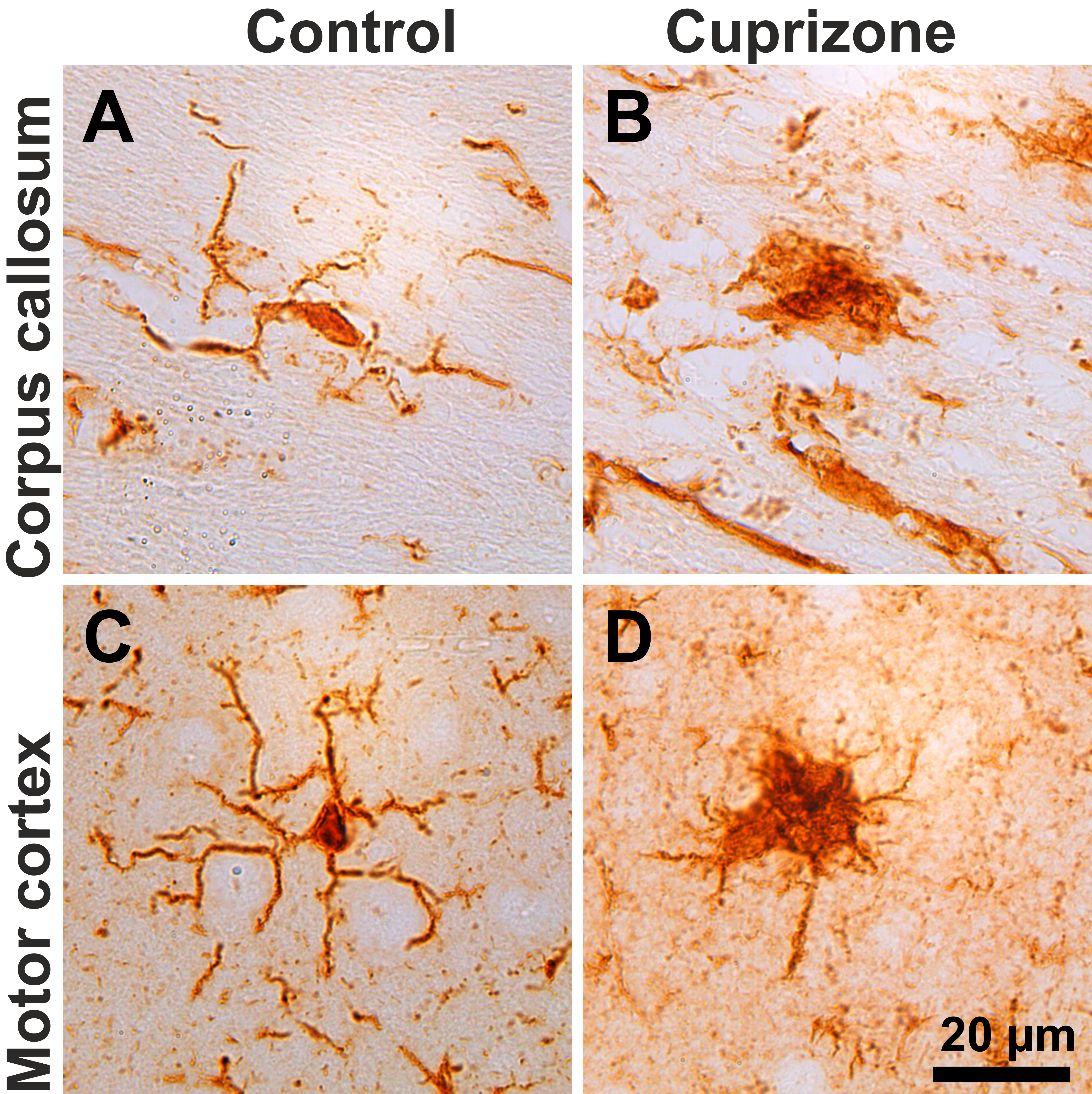

Supplement: Supplementary file 1 [file cells-14-00969-s001.zip › Supplement/Figure S1/Figure S1.tif]
